# Giant adrenal cyst: case study

**Published:** 2010-08-25

**Authors:** C Poiana, M Carsote, P Chirita, D Terzea, S Paun, M Beuran

**Affiliations:** * Carol Davila University of Medicine and Pharmacy, BucharestRomania; ** CI Parhon National Institute of Endocrinology, BucharestRomania; *** Babes National Institute of Research and Development, BucharestRomania; **** Floreasca Emergency Hospital, BucharestRomania

**Keywords:** adrenal cyst, adrenalectomy

## Abstract

One of the rarest situations regarding an adrenal incidentaloma is an adrenal cyst. We present the case of a 61Z–year old male patient diagnosed with peritonitis. During surgery, a right adrenal tumor of 2 cm is discovered. The patient was referred to endocrinology. 6 months later the diameter of the tumor is 7 times bigger than the initial stage. It has no secretory phenotype, except for the small increase of serum aldosterone and the 24–h 17–ketosteroids. Open right adrenalectomy is performed and a cyst of 15 cm is removed. The evolution after surgery is good. The pathological exam reveals an adrenal cyst with calcifications and osteoid metaplasia. The immunohistochemistry showed a positive reaction for CD34 and ACT in the vessels and VIM in the stroma. The adrenal cysts are not frequent and represent a challenge regarding the preoperative diagnostic and surgical procedure of resection. The pathological exam highlights the major aspects.

## Introduction

A gigantic adrenal tumor is a rare finding. In the lack of surgery, the differential diagnosis is difficult. Thus, the surgery becomes an important tool to evaluate the case. The adrenal cysts are exceptional. Because of the hemorrhage, the diameters of the cyst may enlarge rapidly but the clinical picture varies from asymptomatic (incidentaloma discovered by a random ultrasound or CT) to acute complications such as abdominal pain, hypovolemic shock.

## Case presentation

The 61–year old male patient had an appendicle crisis complicated with peritonitis. Surgical approach is performed. During surgery, a right adrenal tumor of almost 2 cm is discovered. After surgery, the patient is referred to an endocrinologist. Six months later the CT scan found a right adrenal tumor of maximum 10 cm and several abdominal lymph nodes. Two more months later, he came to our attention. The clinical exam showed a slightly overweight patient. The biochemical profile pointed mild anemia (hemoglobin – 11.8g/dL, hematocrit – 35.3%). The patient had normal renal and hepatic functions.  The hormonal panel was normal. (Table 1)

**Table 1:** Normal hormonal parameters

Hormone	Value	Normal range
Plasma metanephrines (pg/mL)	10	10 – 90
Plasma normetanephrines (pg/mL)	40	15 – 100
17–OH progesterone (ng/mL)	2.126	0.3 – 3
Serum serotonin (ng/mL)	177	40 – 200
Chromogranin A (pg/mL)	157	40 – 100
Total plasma testosterone (ng/mL)	6.13	2.41 – 8.27

**Figure 1:**
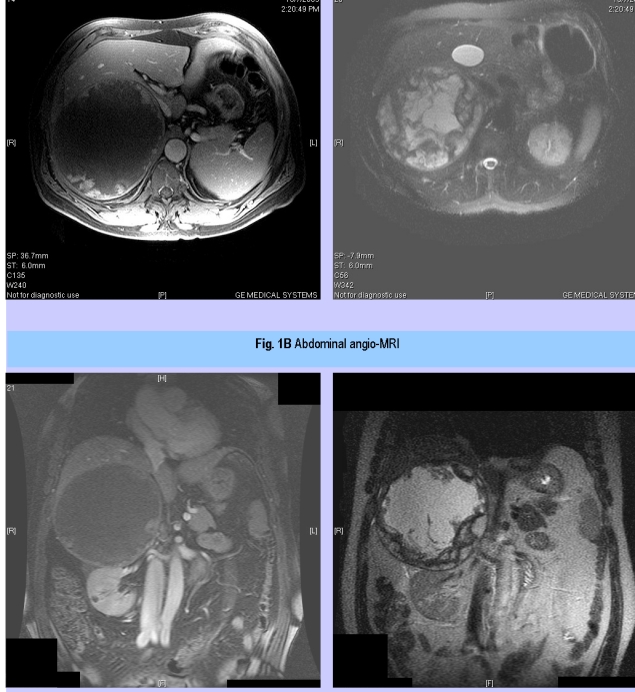
Abdominal angio–MRI: giant adrenal right tumor with liquid areas (A–transversal section, B– axial section)

**Figure 2:**
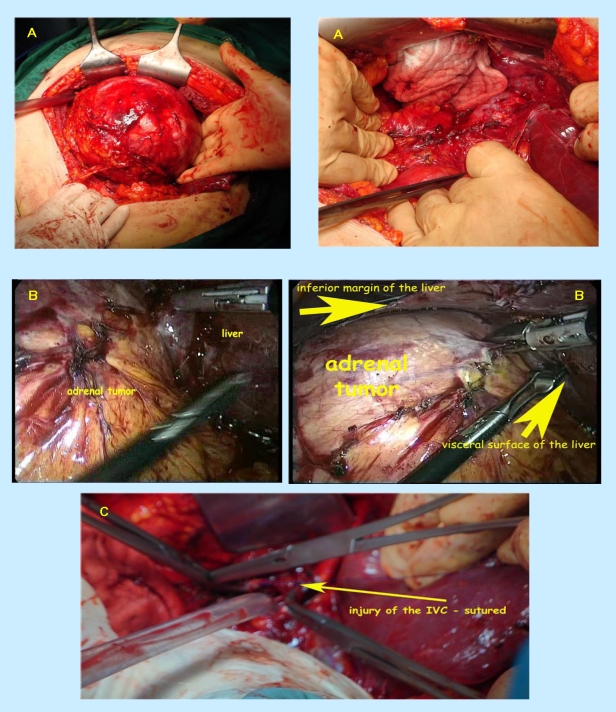
A, B, C  Right adrenalectomy: aspects during surgical procedure

The plasma basal cortisol was of 15.53 mili g/dL (normal between 4.3 and 22.4), with suppression at 0.94 mili g/dL after 2 mg of dexametasone (2 days); plasma basal ACTH – 47.02 pg/mL (normal between 1 and 66). Mildly changed parameters were: serum aldosterone – 278.97 pg/mL (normal between 10 and 160) and 24–hours urinary 17 ketosteroids – 20.2 mg (normal between 8 and 18). The angioMRI revealed a tumor of 15 by 14.7 cm with a large central area filled with liquid signal, an intact capsule in contact with the right kidney, hepatic right lobe, inferior cave vein, preserving a demarcation limit. After the administration of the contrast substance, a loading into the periphery of the tumor took place.  Inter–aorto–cave lymph nodes of 1.8 cm are situated under the left renal hill. (Figure 1) Classical surgical adrenalectomy is performed. No complications were registered. (Figure 2)

**Figure 3:**
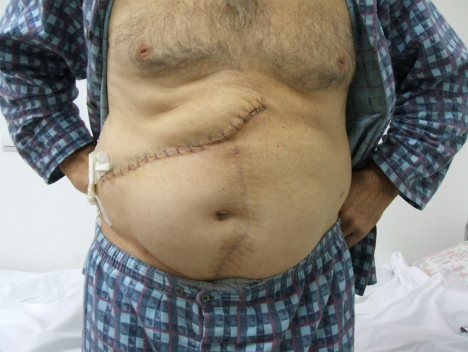
One week after surgery

No adrenal insufficiency was registered. Basal plasma cortisol was of 17 mili g/dL and mild hepatic cytolysis was remitted within 7 days, probably due the hepatic intra–surgery manipulation. The full recovery of the patient was present. The patient will be kept under observation in the next period. (Figure 3)

**Figure 4:**
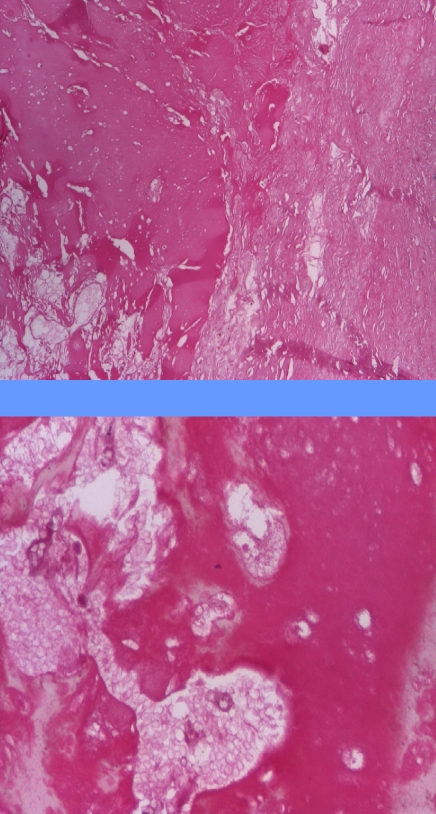
Hematoxilin eosin: different aspects of the adrenal cyst with normal adrenal epithelium

The pathological exam pointed an adrenal cyst having hemorrhagic content, a fibro–conjunctive capsule with areas of calcifications inside the capsule and osteoid metaplasia under the capsule. Normal adrenal tissue was found peri and intra–capsule. (Figure 4) 

**Figure 5:**
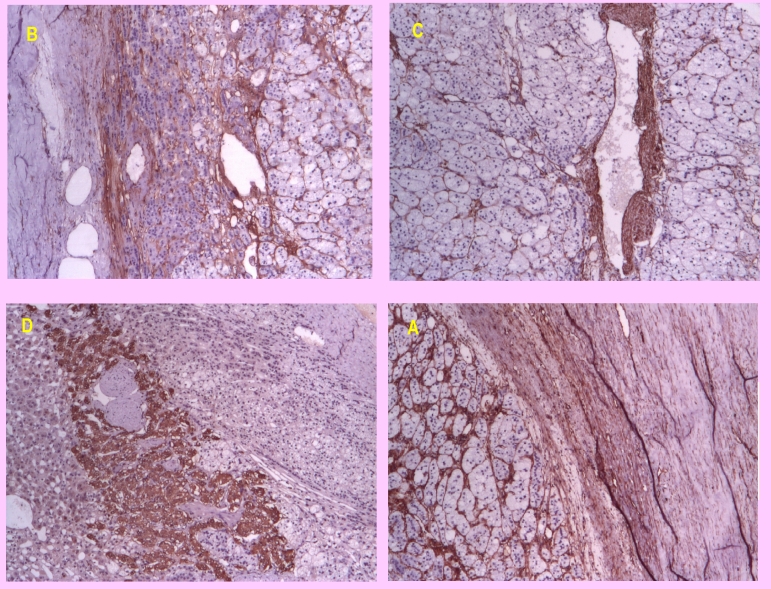
5A–IHC: VIM +ve in the stroma; 5B–IHC: CD34 +ve in the vessels; 5C–IHC: ACT +ve in the vessels; 5D–IHC: CROMO +ve in the medulosuprarenal

The immunohistochemistry revealed stromal positive reaction for VIM, ACT and CD34 positive into the vessels, CROMO positive into the medullar and negative INHIBIN. (Figure 5) 

## Discussions

Adrenal cysts are rare. The reported incidence in clinical series is of 5.4%. [1] In the autopsy series they count less than 0.064 to 0.18%. [2] There are a few similar cases reported in the literature with mixed giant adrenal tumor and rapid increase that suggests an adrenal carcinoma. [3] In this case, the areas of calcification and osteoid metaplasia might suggest a long–standing process but apparently, the tumor increased to 15 cm in 6 months, probably due to the high blood amount. An adrenal carcinoma may also have necrosis and calcification, slightly modified endocrine secretor parameters of the adrenal and rapidly increasing dimensions. There are also a small number of cases in literature regarding adrenal cysts hormonally active (Cushing's syndrome, pheocromocytoma). [1] 

Adrenal cysts are usually asymptomatic; mostly an occasional discovery during ultrasound or CT. In our case, it was a surgical procedure for a different cause. Acute abdominal pain may be presented in some cases. [4] In this case the patient had initially abdominal pain but for another disease. There are some patients reported with sudden onset of abdominal pain and features compatible with acute appendicitis probably caused by a hemorrhage into the cyst. [5] In our case, at the moment of the first surgery, the tumor was small, macroscopically of 2 cm, so a hemorrhage was less probable at that moment. Examples of atypical situations are three cases of acute hemorrhage during pregnancy. [6] Some other emergencies of the cyst are its rupture, acute hypovolemia or shock due to the intense bleeding. [7, 8, 9] Insidious presentation of the cyst is represented by digestive disturbances due to the changes of the local anatomy. While most of the cysts have less than 10 cm, one of the largest described has 45 cm. [10]

From the histological point of view, the adrenal cysts are vascular or endothelial, hemorrhagic or pseudoscyst and epithelial–lined or ‘true’ adrenal cysts. There are a few cases of bilateral cysts reported. [11] 7% of cysts are malignant. [12] Among adrenal cysts, the most common types are epithelial cysts and pseudo–cysts. Some authors appreciate that the adrenal pseudo cyst is the most frequent type. [13] Intra–cystic hemorrhage spontaneous or post–traumatic may be present. This was probably presented in our case, explaining the rapid increase of the tumor. Sometimes, the hemorrhage causes acute anemia that indicates surgery. The operation is usually a laparotomic adrenalectomy, since the laparoscopic approach is not sufficient to control large masses with frequent active bleeding inside. [14] On the other hand, small adrenal cysts do not necessary need surgical approach. They may be followed up by ultrasound or CT and hormonally. [15] There are still controversies related to the adequate management. [16] Surgical indication has tumors larger than 5 cm, symptomatic tumors or rapid expanding. [17] 

A patient with a giant adrenal mass is a challenge regarding the differential diagnosis from other types of adrenal masses. There are many parasitosis with cysts aspects. [18] The diagnosis could only be made following the anatomo–pathological analyses. Pre operative CT guided biopsy (if the tumor is not hormonal active or there is no suspicion of malignancy), urography or angioMRI (like the one in our case) are useful. Complete cyst aspiration, rather than surgical excision, may be carried out initially for diagnosis and management of indeterminate suprarenal cystic lesions and symptomatic cysts of the adrenal gland. [19] The present case demonstrates that the uncommon forms of adrenal masses must be taken into account only after surgery because the pathological evaluation provides the final remark. [20] 

## Conclusion

A giant adrenal cyst mimicking a carcinoma by the preoperative phenotype is a very rare finding. The surgical approach is challenging because of the large tumor dimensions and increased blood amount. Nevertheless, the pathological exam brings the most spectacular aspects regarding the differential diagnosis and the prognosis.
